# Potential protective benefits of Schisandrin B against severe acute hepatitis in children during the COVID-19 pandemic based on a network pharmacology analysis

**DOI:** 10.3389/fphar.2022.969709

**Published:** 2022-08-11

**Authors:** Yanhua Fang, Lingling Zhang, Zhe Wang, Ruoyu Wang, Shanshan Liang

**Affiliations:** ^1^ The Key Laboratory of Biomarker High Throughput Screening and Target Translation of Breast and Gastrointestinal Tumor, Affiliated Zhongshan Hospital of Dalian University, Dalian, Liaoning, China; ^2^ Oncology Department, Affiliated Zhongshan Hospital of Dalian University, Dalian, Liaoning, China

**Keywords:** hepatitis, adenovirus, SARS-CoV-2, Schisandrin B, network pharmacology

## Abstract

**Aims:** Reports of hepatitis in children during the coronavirus disease 2019 (COVID-19) pandemic garnered worldwide attention. The most probable culprits are adenovirus and severe acute respiratory syndrome coronavirus-2 (SARS-CoV-2). At present, the optimal symptomatic treatment consists of a combination of anti-COVID-19 and hepatitis symptom alleviators. Schisandrin B (SchB) has been known to have liver-protective properties for a long time, whereas anti-COVID-19 properties only recently have been discovered. In the case of COVID-19 with hepatitis of unknown origin, we used network pharmacology to explore the symptomatic therapy and protective effects of SchB.

**Main methods:** The most probable protein targets of SchB were predicted in the SwissTargetPrediction database. The GeneCards, National Center for Biotechnology Information, and Online Mendelian Inheritance in Man databases were used to compile information on the diseases hepatitis, adenovirus, and SARS-CoV-2. Following the use of a Venn diagram viewer to identify intersection genes, we constructed a protein–protein interaction network and identified the core genes. Gene Ontology and Kyoto Encyclopedia of Genes and Genomes enrichment, as well as molecular docking, were employed to highlight the mechanisms of SchB on hepatitis.

**Key findings:** SchB contains 27 targets on adenovirus_hepatitis and 16 targets on SARS-CoV-2_hepatitis, with 12 shared genes. Both target populations clustered in viral infection and cancer pathways, as well as in processes such as kinase activity phosphatase, cell adhesion, and ATPase binding. These genes might be closely related to liver damage and membrane binding from adenovirus or SARS-CoV-2 infections. In addition, epidermal growth factor receptor, HSP90AA1, and MAPK1 were among the top five targets of both SchB SARS-CoV-2 hepatitis and SchB adenovirus hepatitis.

**Significance:** SchB may target common protective targets and mechanisms against acute hepatitis caused by adenovirus or by SARS-CoV-2 in children during the COVID-19 pandemic. These findings indicate SchB’s potential as a treatment for hepatitis of unknown origin.

## Introduction

The coronavirus disease 2019 (COVID-19), which is caused by the severe acute respiratory syndrome coronavirus-2 (SARS-CoV-2), poses a significant threat to global public health, endangering economic and social stability and necessitating the rapid development of novel therapies and preventive measures (Hu et al., 2021; Hossain et al., 2022; Islam et al., 2022). Even worse, several counties and regions have reported more than 600 cases of pediatric hepatitis of unknown origin in May 2022, with a clear connection to the COVID-19 outbreak. Hepatitis typically is related to viral infections (including hepatitis viruses A, B, C, D, and E) and alcohol and drug use. In this study, none of the five hepatitis viruses were found in any of the reported patients. Because the majority of affected children were not vaccinated against COVID-19, vaccines also can be ruled out as a possible cause (Mohapatra et al., 2022). In addition, alcohol- and drug-related reasons were ruled out, given that most children were previously healthy and did not consume alcohol. At present, the cause of hepatitis is unknown, but adenovirus and SARS-CoV-2 are the most commonly cited and suspected causative agents. At first, inquiry reports were issued with extreme caution, with the major possibilities being adenovirus or a novel SARS-CoV-2 variant, in addition to the possibility of an undiagnosed virus (Marsh et al., 2022). Then, some reports favored adenovirus infection as the cause (Baker et al., 2022), whereas others suggested that SARS-CoV-2 infection was the culprit (Antala et al., 2022; Nishiura et al., 2022). At present, a study investigated SARS-CoV-2 as superantigens, and adenovirus might serve as an accomplice (Brodin and Arditi, 2022). Before publishing this article, the most recent study stated that clinical data are required to determine the underlying causes of these patients’ illnesses and to evaluate any possible associations with adenovirus. It also highlighted the SARS-CoV-2 infection both before and after the patient’s illness began (Cates et al., 2022). At present, anti-COVID-19 and symptom-relieving hepatitis medications are the most effective means to treat both prevention and symptoms.

**FIGURE 1:**
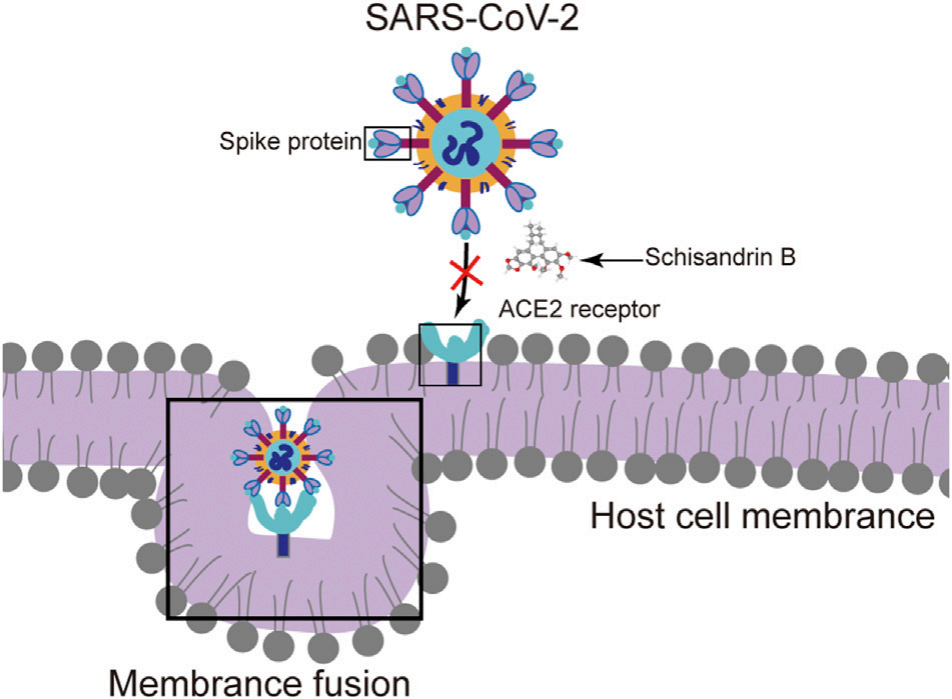
Schisandrin B (SchB) inhibits SARS-CoV-2 infection. SchB inhibits SARS-CoV-2 virus infection by blocking spike (S) protein-mediated membrane fusion.

Schisandrin B (SchB), a natural small molecule compound isolated from *Schisandra chinensis* (*Turcz.*) *Baill.*, has been reported widely to afford hepatoprotection against oxidative stress, inflammation, and anticarcinogenic activities (Wu et al., 2004; Stacchiotti et al., 2009; Zhu et al., 2012; Li et al., 2014). In recent years, many natural substances have been demonstrated to hold biological significance (Rahman et al., 2022c), including antiviral properties, as well as potential application in the treatment of COVID-19 (Islam et al., 2021). Additional research noted that SchB had an antiviral effect that was resistant to human immunodeficiency virus reverse transcriptase (Xu et al., 2015). SchB was evaluated as an effective SARS-CoV-2 spike (S) pseudo-type virus inhibitor in the absence of particular medications during the COVID-19 outbreak by inhibiting S-protein-mediated membrane fusion. Further experiments confirmed that SchB inhibited authentic SARS-CoV-2 with a highly selective index (Cao J. et al., 2022) (Figure 1). Based on these findings, we suggest that SchB may have a protective effect against this unknown origin hepatitis.

**FIGURE 2:**
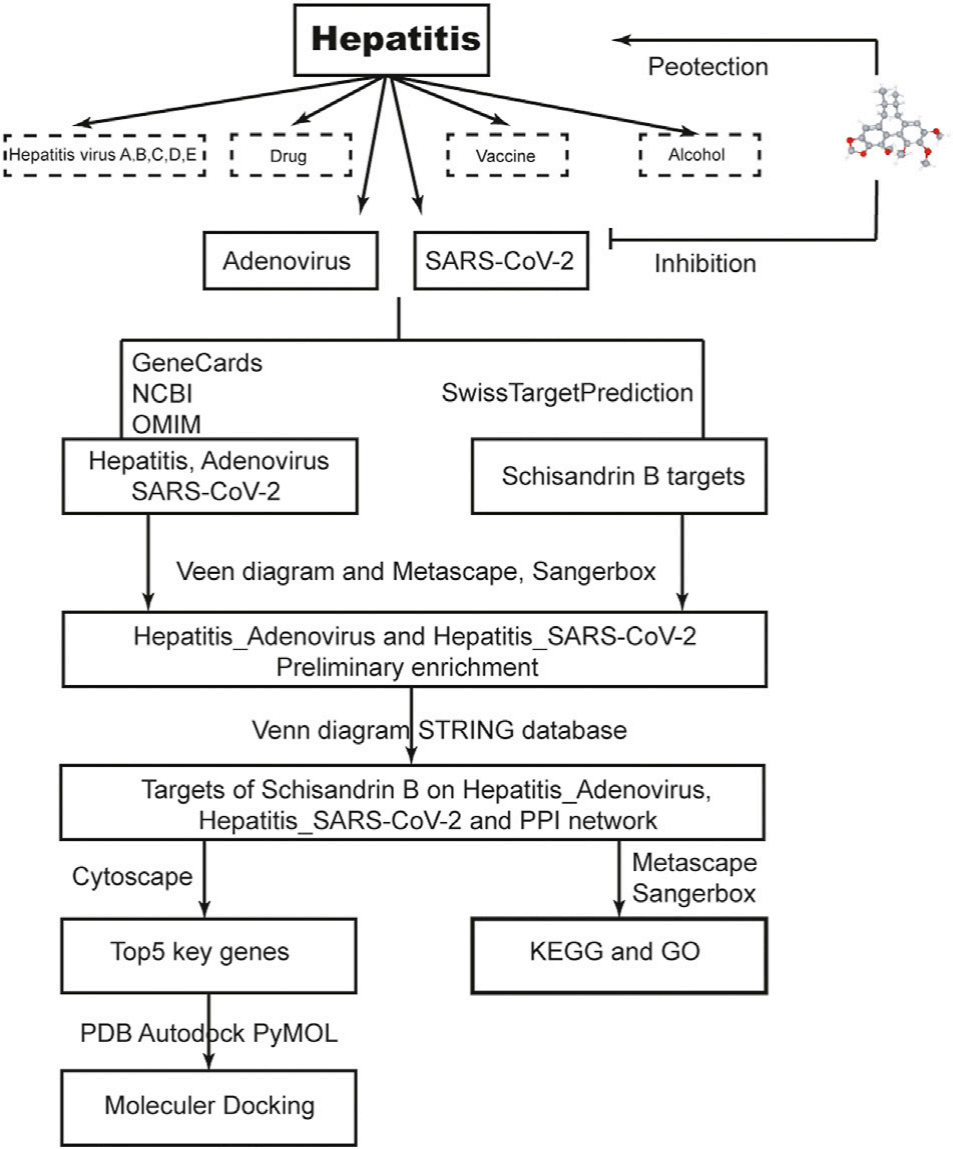
Workflow of the study. The flow diagram indicates the antiviral action and mechanism of SchB against adenovirus_hepatitis and SARS-CoV-2_hepatitis using the network pharmacology analysis approach.

Network pharmacology employs computers and high throughput omics data analysis to investigate the pharmacological mechanism and active components of traditional Chinese medicine (TCM). The experimental workload can be significantly reduced when the target prediction results match the experimental verification (Cao X. et al., 2022). This method has been used to assess certain promising TCM and Indian Ayurvedic medications for the most difficult lung disorders, particularly SARS-CoV-2 (Rahman et al., 2022a) or other diseases (Rahman et al., 2022b). In this study, we used a series of network pharmacology analyses to understand and characterize pharmacological targets, biological functions, binding capacity, and therapeutic mechanisms of SchB in hepatitis that are related to adenovirus or SARS-CoV-2. The flow chart of this study is shown in Figure 2.

## Materials and methods

### Prediction of putative targets in Schisandrin B

SchB’s two-dimensional (2D) structure and simplified molecular input line entry system (SMILES) metadata were obtained from the PubChem database (https://pubchem.ncbi.nlm.nih.gov/). Once SMILES data were entered into the SwissTargetPrediction database, we selected only those targets labeled “homo sapiens” for additional research (http://www.swisstargetprediction.ch/).

### Identification of Schisandrin B targets in adenovirus/SARS-CoV-2 and hepatitis

On 11 May 2022, the target genes for adenovirus, SARS-CoV-2, and hepatitis were extracted from three databases: GeneCards (https://www.genecards.org/) with a relevance score of >1.0, Online Mendelian Inheritance in Man (OMIM) (https://omim.org/), and the National Center for Biotechnology Information (NCBI) (https://www.ncbi.nlm.nih.gov/). The search terms “hepatitis,” “adenovirus,” and “SARS-CoV-2” were used to find “homo sapiens” targets from the available platforms. The intersection between SchB potential targets and adenovirus/SARS-CoV-2–linked hepatitis genes was determined using a Venn diagram viewer (Bardou et al., 2014).

### Construction of protein–protein interaction and key module network

The Search Tool of Retrieval of Interacting Genes (STRING) (http://string-db.org/) database was employed to create a protein–protein interaction (PPI) systematic network and visualized the result. The network’s disconnected nodes were concealed, and shared proteins with a minimum interaction score of 0.9 were selected. To construct the PPI network, we obtained the STRING tsv file and imported the file into Cytoscape version 3.8.1. We used CytoHubba to examine the module network and selected the five genes with the highest score.

### Enrichment of Schisandrin B targets on adenovirus_hepatitis and SARS-CoV-2_hepatitis

To further understand the processes underlying SchB’s effectiveness, we conducted a gene enrichment analysis of the screened SchB targets in adenovirus/SARS-CoV-2 and hepatitis. We used Metascape (http://metascape.org/gp/index.html#/main/step1) to perform both Kyoto Encyclopedia of Genes and Genomes (KEGG) pathway enrichment and Gene Ontology (GO) biological functional enrichment. The enrichment backdrop was made up of genes chosen by the STRING database. Based on their membership similarities, terms with a *p*-value of <0.01, a minimum count of 3, and an enrichment factor of >1.5 were sorted into clusters. We performed data visualization as Bubble Diagrams using the online platform Sangerbox (http://sangerbox.com/Tool).

### Molecular docking

The crystal structures of the top five targets were obtained from the Program DataBase (PDB) (http://www.rcsb.org/), and the PDB format of SchB was obtained from the PubChem database. We used AutoDock 4.2.6 and PyMOL to pretreat the active ingredients and targets. AutoDock-Tools 1.5.7 software performed the docking tests between SchB and key targets, followed by mapping of the lowest energy binding mode using PyMOL.

## Results

### Acquisition and Kyoto Encyclopedia of Genes and Genomes analysis of intersection genes between hepatitis and adenovirus/SARS-CoV-2

**FIGURE 3:**
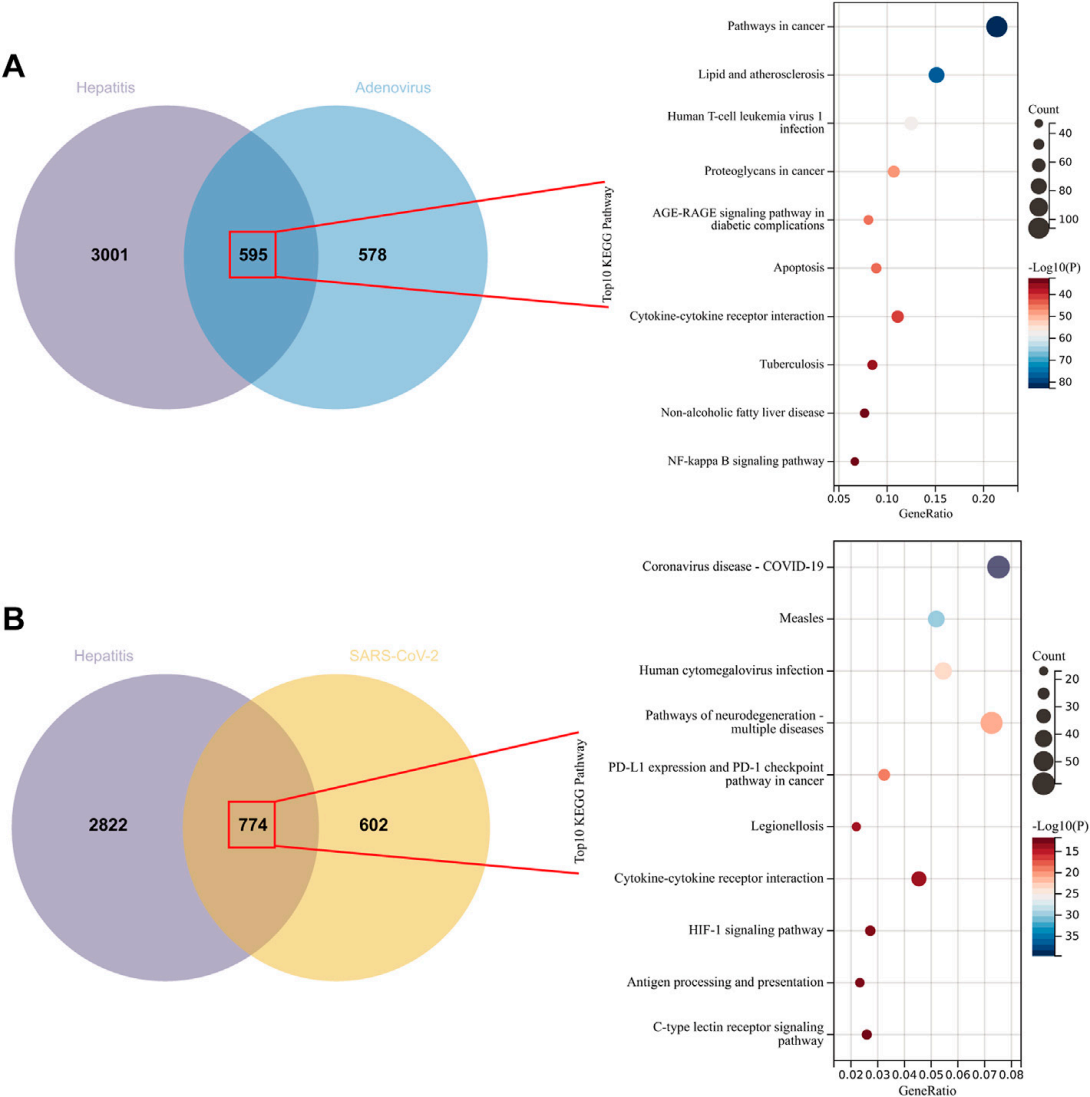
Intersection genes between hepatitis and adenovirus/SARS-CoV-2. **(A)** Venn diagram representing the 595 intersecting genes of hepatitis with Adenovirus (left). Kyoto Encyclopedia of Genes and Genomes (KEGG) analysis revealed that they are related to liver metabolism, viral infection, receptor interaction, and immune-inflammatory response (right). **(B)** Venn diagram representing the 774 intersecting genes of hepatitis with SARS-CoV-2 (left), and they enriched in viral infection, receptor interaction, and immune-inflammatory response (right).

Network pharmacology extracted 3597, 1173, and 1376 genes from the GeneCards, OMIM, and NCBI databases, respectively, which were linked with hepatitis, adenovirus, and SARS-CoV-2. At first, we wanted to determine which genes were implicated in adenovirus_hepatitis and SARS-CoV-2_hepatitis, as well as the pathways in which these genes were enriched. We identified 595 and 774 intersection genes in adenovirus_hepatitis and SARS-CoV-2_hepatitis, respectively (Figure 3). Subsequent KEGG enrichment analysis revealed that the genes in adenovirus_hepatitis participated mostly in the following pathways: cancer, lipid and atherosclerosis, human T-cell leukemia virus 1 infection, AGE-RAGE signaling in diabetic complications, cytokine-cytokine receptor interaction, nonalcoholic fatty liver disease, and NF-kappa B (Figure 3A). The pathways are associated with liver metabolism, viral infection, receptor interaction, and immune-inflammatory response. Genes in SARS-CoV-2_hepatitis were related primarily to COVID-19, measles, human cytomegalovirus (HCV) infection, neurodegeneration multiple diseases, PD-L1 expression, and the PD-1 checkpoint pathway in cancer, legionellosis, cytokine-cytokine receptor interaction, antigen processing and presentation, and C-type lectin receptor pathway (Figure 3B). They were, in summary, associated with viral infection, receptor interaction, and immunological inflammatory response.

### Acquisition of Schisandrin B targets on adenovirus_hepatitis and SARS-CoV-2_hepatitis

**FIGURE 4:**
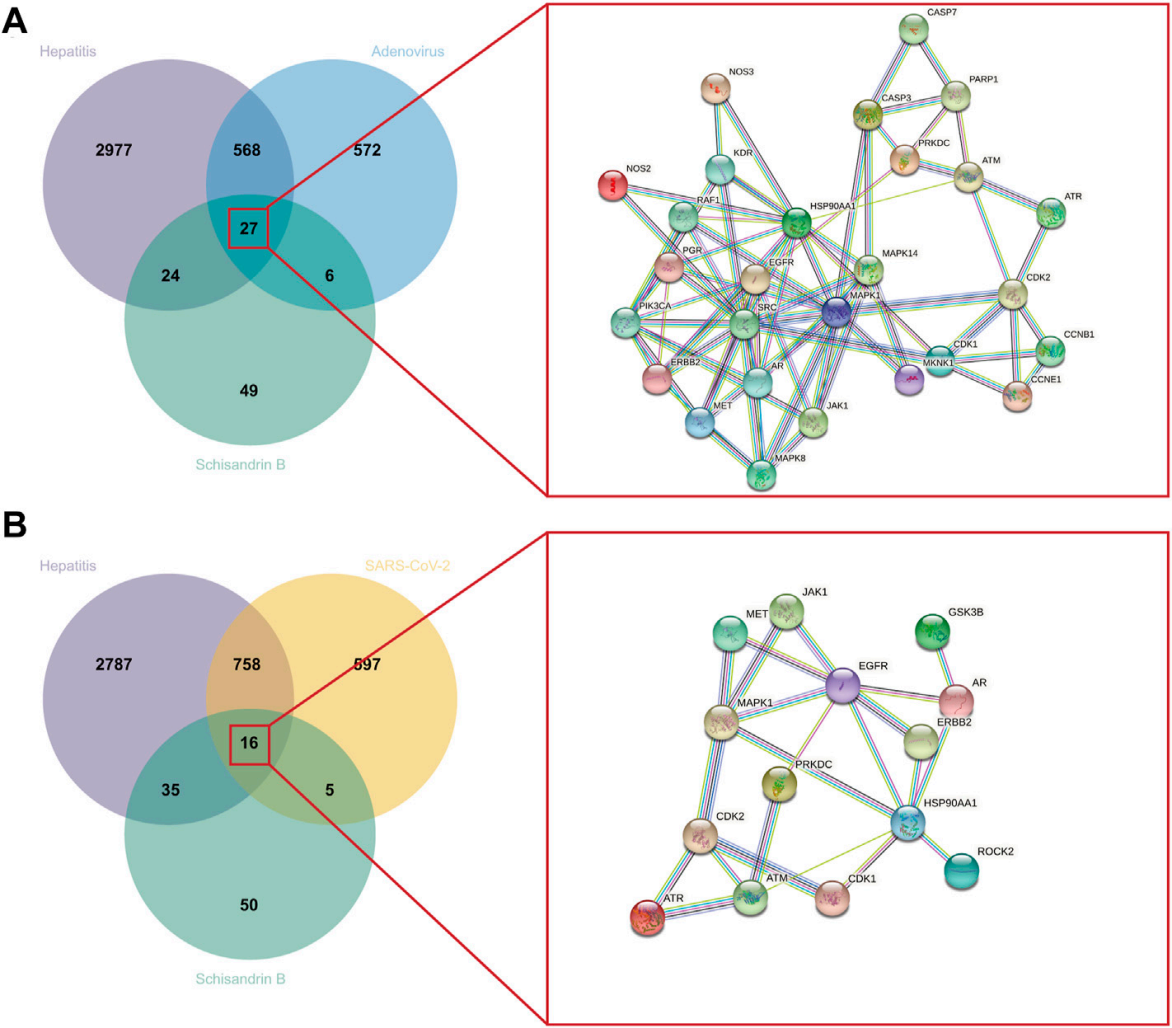
Identification of SchB targets on adenovirus/SARS-CoV-2_hepatitis. **(A)** Venn diagram representing the 27 intersecting targets of SchB with adenovirus_hepatitis (left). A PPI network was constructed with these common genes, which met the conditions interaction score of >0.9, and disconnected nodes were hidden (right). **(B)** Venn diagram representing the 16 intersecting targets of Schisandrin B with SARS-CoV-2_hepatitis (left), and the PPI network met the above filter criteria (right).

SwissTargetPrediction provided 106 SchB-associated targets for “homo sapiens.” Adenovirus_hepatitis and SARS-CoV-2_hepatitis genes overlapped with SchB targets, resulting in the 27 and 16 intersection genes of SchB against adenovirus_hepatitis and SARS-CoV-2_hepatitis, respectively. We constructed the PPI networks by submitting overlapping genes to the STRING database while hiding the free point (ALOX5, TRPV1) (Figure 4). Table 1 compares SchB targets on adenovirus_hepatitis and SARS-CoV-2_hepatitis (hiding the two free points). They shared 12 genes in common, accounting for 86% of the SARS-CoV-2_hepatitis collection.

**TABLE 1:** Comparison of Schisandrin B (SchB) targets on adenovirus/SARS-CoV-2_hepatitis.

Adenovirus_hepatitis	SARS-CoV-2_hepatitis	Target name
MET	MET	Tyrosine-protein kinase MET
MAPK1	MAPK1	MAP kinase ERK2
ATM	ATM	Serine-protein kinase ATM
ERBB2	ERBB2	Receptor protein-tyrosine kinase erbB-2
CDK1	CDK1	Cyclin-dependent kinase 1
AR	AR	Androgen receptor
CDK2	CDK2	Cyclin-dependent kinase 2
ATR	ATR	Serine-protein kinase ATR
HSP90AA1	HSP90AA1	Heat shock protein HSP 90-alpha (by homology)
PRKDC	PRKDC	DNA-dependent protein kinase
EGFR	EGFR	Epidermal growth factor receptor erbB1
JAK1	JAK1	Tyrosine-protein kinase JAK1
—	ROCK2	Rho-associated protein kinase 2
—	GSK3B	Glycogen synthase kinase-3 beta
CASP7	—	Caspase-7
CCNE1	—	Cyclin-dependent kinase 2/cyclin E
CCNB1	—	Cyclin-dependent kinase 1/cyclin B
MKNK1	—	MAP kinase-interacting serine/threonine-protein kinase MNK1
PIK3CA	—	PI3-kinase p110-alpha subunit
CASP3	—	Caspase-3
KDR	—	Vascular endothelial growth factor receptor 2
MAPK14	—	MAP kinase p38 alpha
MAPK8	—	c-Jun N-terminal kinase 1
NOS2	—	Nitric oxide synthase, inducible
NOS3	—	Nitric-oxide synthase, endothelial
PARP1	—	Poly [ADP-ribose] polymerase-1
PGR	—	Progesterone receptor
RAF1	—	Serine/threonine-protein kinase RAF
SRC	—	Tyrosine-protein kinase SRC

### Identification of core targets

**FIGURE 5:**
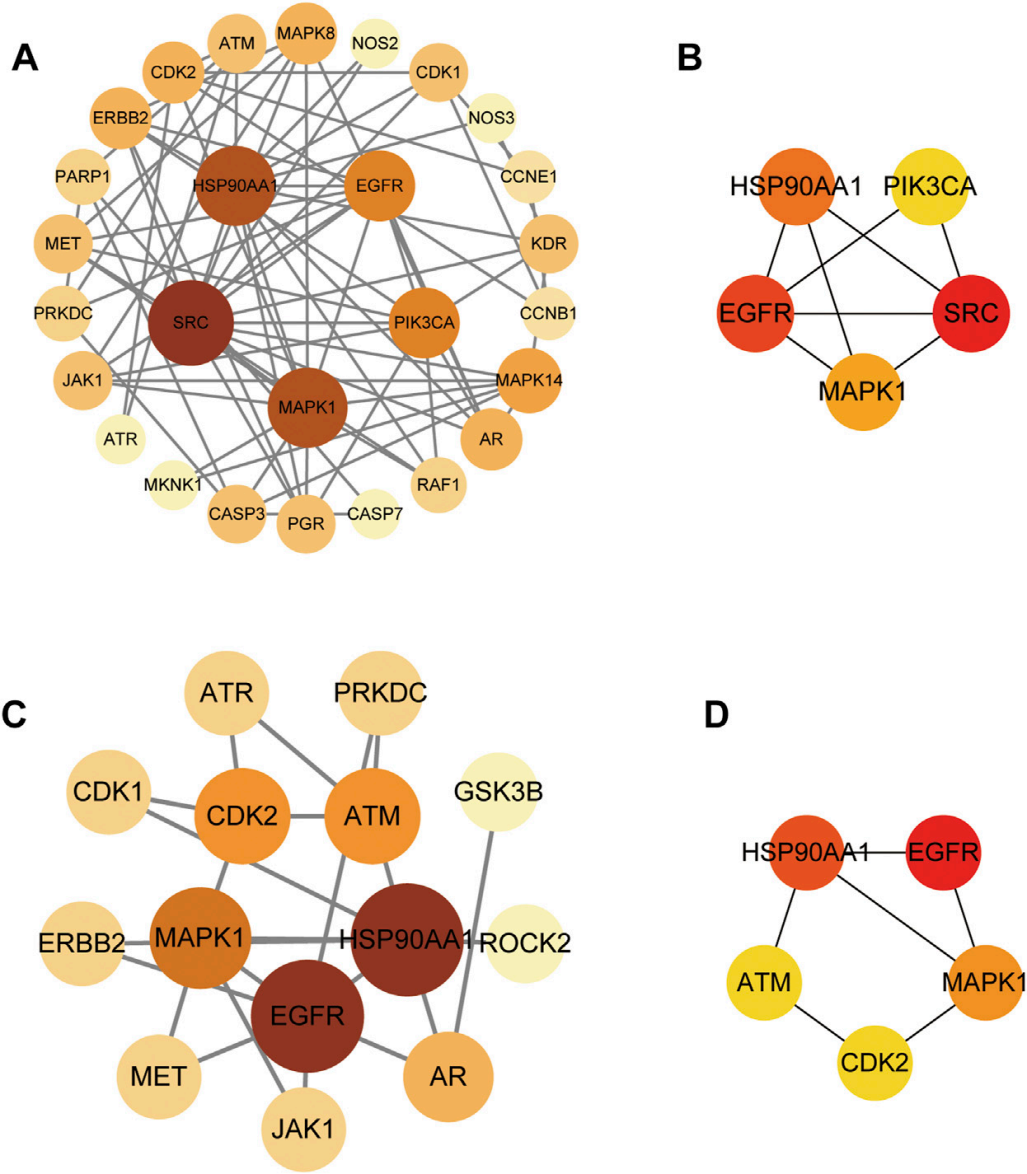
Protein–protein interaction (PPI) network and module analysis network. **(A)** PPI network of SchB targets on adenovirus_hepatitis presented by Cytoscape v3.8.2. **(B)** Module analysis of the CytoHubba plug-in identified the top five genes to construct the key module network of **(A)**. **(C)** PPI network of SchB targets on SARS-CoV-2_hepatitis presented by Cytoscape v3.8.2. **(D)** CytoHubba identified the top five genes of **(C)**.

We conducted further PPI analysis to screen core targets by Cytoscape (Figure 5). The PPI network of SchB against adenovirus_hepatitis included 27 nodes and 75 edges (Figure 5A). Module analysis of the CytoHubba plug-in identified the top five genes to construct a key module network, which included five nodes and eight edges. The top five core targets were SRC, epidermal growth factor receptor (EGFR), HSP90AA1, MAPK1, and PIK3CA (Figure 5B; Table 2). SchB’s PPI network against SARS-CoV-2_hepatitis had 14 nodes and 22 edges (Figure 5C). EGFR, HSP90AA1, MAPK1, CDK2, and ATM were among the top five genes identified using CytoHubba (Figure 5D; Table 3). The three core targets they shared in common were EGFR, HSP90AA1, and MAPK1.

**TABLE 2:** Top five targets with potentially critical roles in SchB treatment of adenovirus_hepatitis.

Gene abbreviation	Degree	Closeness centrality	Betweenness centrality
SCR	14.0	19.5	98.3
EGFR	9.0	16.8	44.2
HSP90AA1	12.0	18.8	132.6
MAPK1	12.0	19.0	157.1
PIK3CA	9.0	15.8	17.8

**TABLE 3:** Top five targets with potentially critical roles in SchB treatment of SARS-CoV-2_hepatitis.

Gene abbreviation	Degree	Closeness centrality	Betweenness centrality
EGFR	7.0	9.8	41.5
HSP90AA1	7.0	10.0	64.2
MAPK1	5.0	8.8	24.7
CDK2	4.0	7.8	14.7
ATM	4.0	8.0	21.5

### Kyoto Encyclopedia of Genes and Genomes pathway and Gene Ontology functional annotation analysis

**FIGURE 6:**
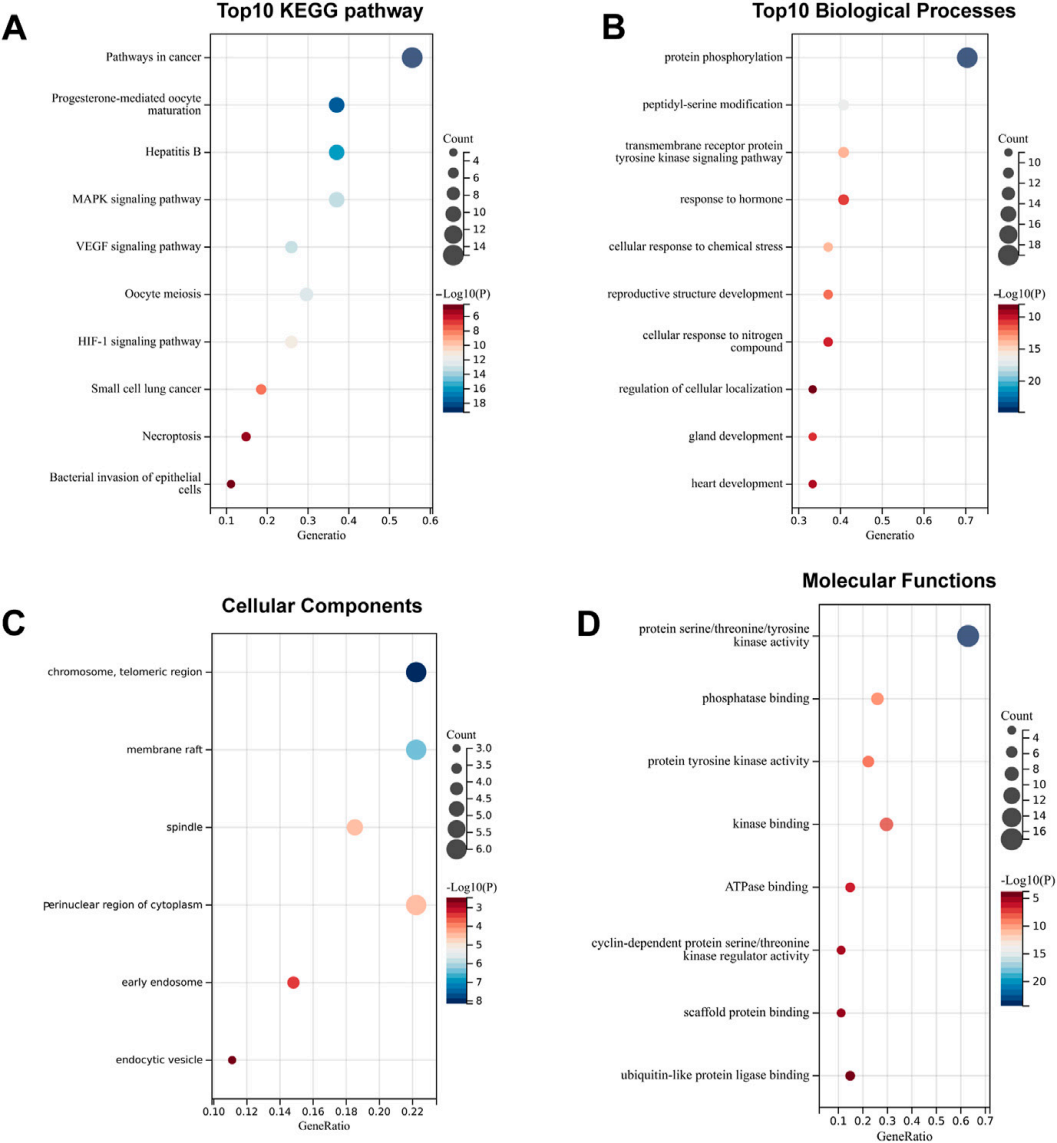
Functional analysis of SchB targets on adenovirus_hepatitis. **(A)** The top 10 enriched KEGG pathways of genes that SchB targets on adenovirus_hepatitis. **(B–D)** The top 10 enriched terms of biological process, cellular component, and molecular function.

**FIGURE 7:**
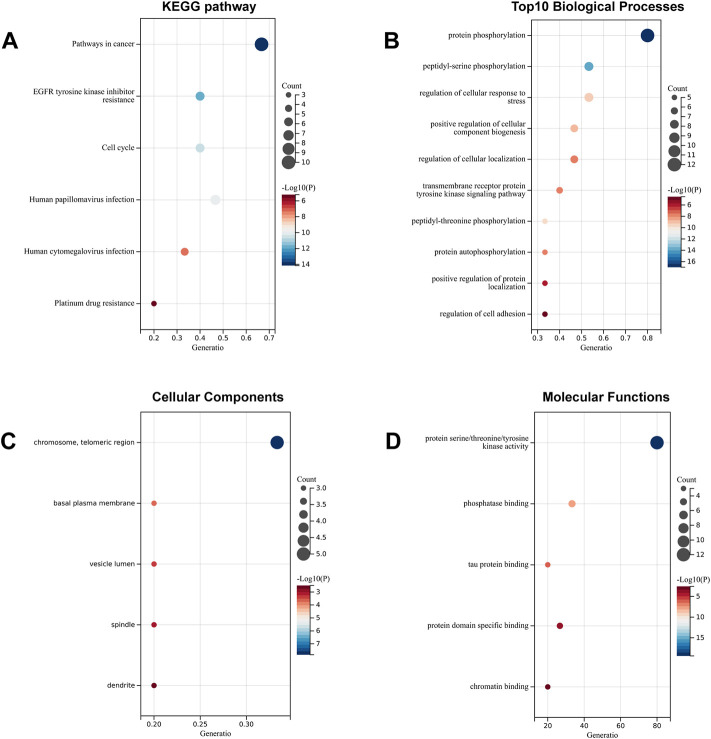
Functional analysis of SchB targets on SARS-CoV-2_hepatitis. **(A)** The enriched KEGG pathways of genes SchB targets on SARS-CoV-2_hepatitis. **(B–D)** The top 10 enriched terms of biological process, cellular component, and molecular function.

To systematically elucidate the multiple mechanisms of SchB in the treatment of adenovirus-related hepatitis or SARS-CoV-2–related hepatitis, we conducted KEGG and GO enrichment analyses of the genes SchB against adenovirus/SARS-CoV-2_hepatitis, which was obtained according to the STRING database analysis. We identified the top 10 pathways based on the number of enriched genes and the *p*-value of SchB’s KEGG pathway against adenovirus_hepatitis (Figure 6A). These pathways were highly correlated with cancer, progesterone-mediated oocyte maturation, hepatitis B, and MAPK pathways. According to the GO enrichment analysis of SchB against adenovirus_hepatitis (Figures 6B–D), the targets were closely linked to protein phosphorylation, transmembrane receptor protein tyrosine kinase, membrane raft, spindle, protein serine/threonine/tyrosine kinase activity, phosphatase binding, kinase binding, ATPase binding, and scaffold protein binding. The KEGG pathway analysis of SchB against SARS-CoV-2_hepatitis demonstrated a correlation with cancer, EGFR tyrosine kinase inhibitor resistance, cell cycle, human papillomavirus infection, HCV infection, and platinum drug resistance (Figure 7A). The GO enrichment showed that these genes were concentrated in protein phosphorylation, regulation of cellular response to stress, regulation of cell adhesion, chromosome telomeric region, basal plasma membrane, vesicle lumen, spindle, dendrite, kinase activity, phosphatase binding, tau protein binding, protein domain-specific binding, and chromatin binding (Figures 7B–D).

### Validation of Schisandrin B–target interaction by molecular docking

**FIGURE 8:**
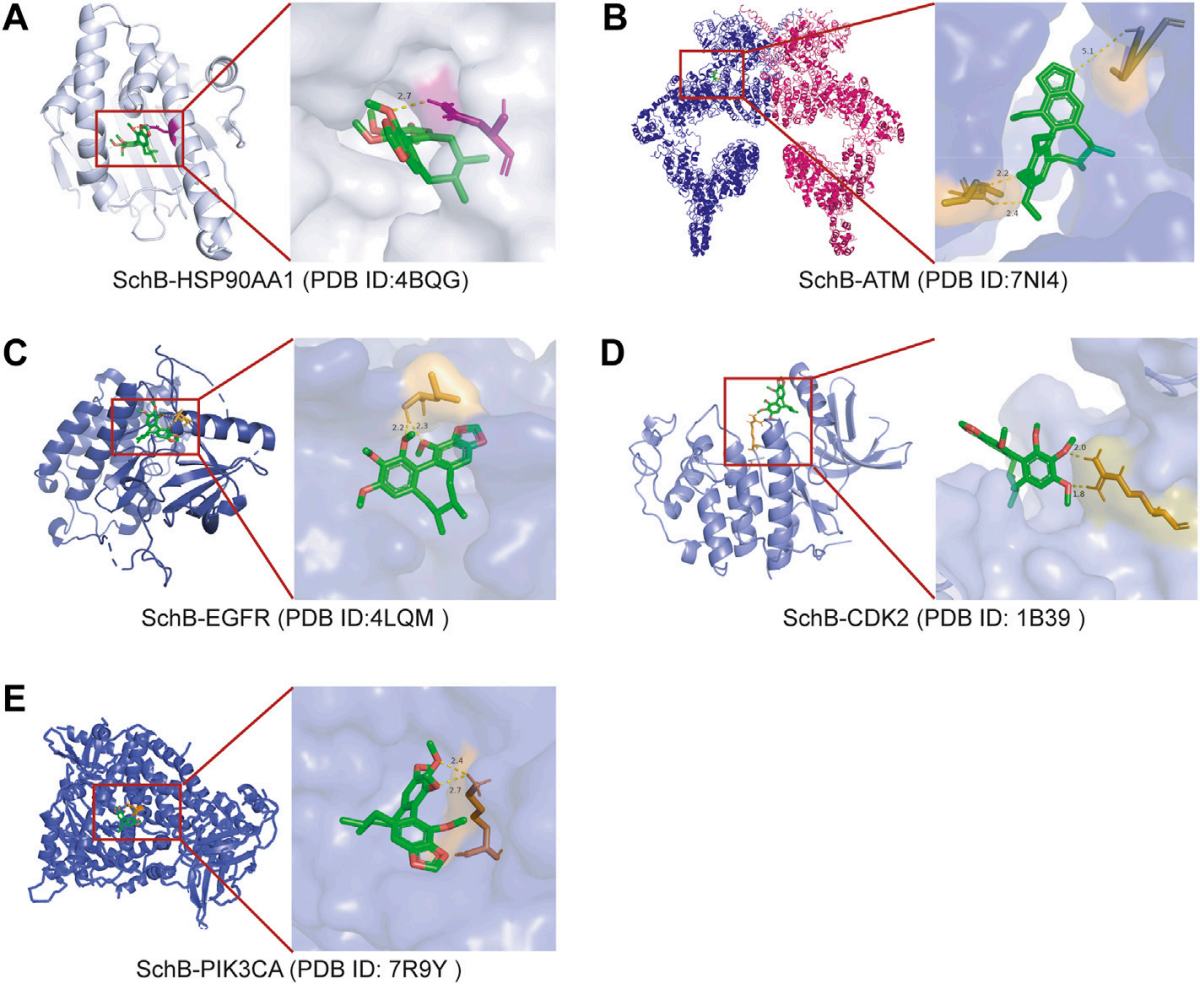
Molecular models of SchB binding to the most probable binding targets. Proteins **(A)** HSP90AA1, **(B)** ATM, **(C)** EGFR, **(D)** CDK2, and **(E)** PIK3CA are shown interacting with a SchB molecule. The binding amino acidic sites and other details are shown on the right.

The molecular docking analysis visually showed the interaction between SchB and its potential protein targets associated with adenovirus/SARS-CoV-2_hepatitis (Figure 8). Schematic diagrams of drug-target binding mode are displayed on the left, and the details are on the right of the figure. Protein structures of the core targets were available on the PDB database (HSP90AA1, ID:4BQG; ATM, ID:7NI4; EGFR, ID:4LQM; CDK2, ID: 1B39; and PIK3CA, ID: 7R9Y). The results showed that SchB formed a hydrogen bond with ASU-51 of HSP90AA1 (Figure 8A), and the binding affinity was −7.55 kcal/mol. SchB combined ATM with ILE-1927, PRO-2842, and ASP-2841 (Figure 8B), and the binding affinity was −5.85 kcal/mol. Similar SchB binding affinity was observed between EGFR (−4.95 kcal/mol) and CDK2 (−4.84 kcal/mol), and the amino acid residue was LEU-703 and ARG-126, respectively (Figures 8C, D). The binding domain of PIK3CA was LYS-672 (Figure 8E) with a binding affinity of −4.8 kcal/mol. Among these interactions, HSP90AA1 and ATM exhibited significantly lower binding energy (Table 4).

**TABLE 4:** Different binding energies and binding acid amine of SchB polysulfides with five selected targets.

Rank	Target	Uniprot ID	Affinity (kcal/mol)	Binding acid amine
1	HSP90AA1	P07900	−7.55	ASU-51
2	ATM	Q13315	−5.85	ILE-1927, PRO-2842, ASP-2841
3	EGFR	P00533	−4.95	LEU-703
4	CDK2	P24941	−4.84	ARG-126
5	PIK3CA	P42336	−4.8	LYS-672

## Discussion

SchB, a dibenzocyclooctadiene lignan and one of the principal active components of *S. chinensis*, has demonstrated hepatoprotective, anti-inflammatory, and antioxidant effects. The dried fruits of *S. chinensis* and their extracts have been shown to aid neurological, gastrointestinal, and cardiovascular conditions as well as to protect against mitochondrial malfunction, insomnia, and liver damage (Stacchiotti et al., 2009; Kopustinskiene and Bernatoniene, 2021). We conducted the current analysis in the context of an outbreak of pediatric hepatitis of unknown origin during the COVID-19 pandemic, taking into account SchB’s hepatoprotective action and reviewing the most recent literature to determine its possible anti-SARS-CoV-2 effect. As a result, we used a network pharmacological technique to predict the possible protective mechanisms of SchB against pediatric hepatitis of unknown origin, providing the framework for future research.

Available studies show that SchB protects against nonalcoholic fatty liver disease and hepatic cirrhosis and prevents liver cancer (Leong and Ko, 2016). The protection mechanisms that have been uncovered in this approach are as follows: 1) SchB can protect the liver by modulating Nrf2-mediated antioxidant pathway and NF-kB-mediated proinflammatory cascade (ERK, ROS, TNF-a, and IL-6) (Li et al., 2008; Leong and Ko, 2015); 2) Gomisin N, a stereoisomer of SchB, ameliorated hepatic steatosis against ER stress by reducing the expression of ER markers Grp78, CHOP, and XBP1 and by activating AMP-activated protein kinase (AMPK) (Jang et al., 2016; Yun et al., 2017); and 3) SchB inhibited hepatoma cell proliferation and induced apoptosis through the caspase-3 pathway accompanied by the downregulation of Hsp70 protein expression (Wu et al., 2004). Although minimal antiviral research has been conducted on SchB, direct evidence indicates that it can resist SARS-CoV-2 by reducing cell binding (Cao J. et al., 2022). Furthermore, *S. chinensis* and its extracts have been proven to have resistance to different viruses. For example, Schisandrin A inhibited dengue viral replication, and bicyclol (an analog of Schisandrin C) inhibited chronic viral hepatitis B and C (Yu et al., 2017). Bicyclol has been used widely to treat chronic hepatitis B virus (HBV) and HCV in China (Liu, 2009), which further leads us to believe that SchB may have a therapeutic effect on virus-related hepatitis.

Because the etiology of children’s hepatitis of unknown origin is uncertain, we examined two causal factors: adenovirus and SARS-CoV-2. We discovered that many of the genes in SchB_adenovirus_hepatitis and SchB_SARS-CoV-2_hepatitis overlapped, and three of the five core targets screened in each set were the same (i.e., EGFR, HSP90AA1, and MAPK1). As a result, independent of the viral causative agents (adenovirus or SARS-CoV-2), SchB may have a similar protective effect against hepatitis. These genes were KEGG enriched proximally, and the two sets were compared. The majority of the genes in both collections were enriched in cancer-related pathways. In addition, SchB_adenovirus_hepatitis was also enriched in the hepatitis B pathway, and SchB_SARS-CoV-2_hepatitis was enriched in the human papillomavirus, HCV infection, and cell cycle pathways. The focus of the enrichment was on cancer rather than on hepatitis or viral infection pathways, which was unexpected.

We then backtracked the genes enriched on the cancer pathway in both collections. Collection of the adenovirus-related genes contained AR, CASP3, CASP7, CCNE1, CDK2, EGFR, ERBB2, HSP90AA1, JAK1, MET, NOS2, PIK3CA, MAPK1, MAPK8, RAF1, KDR, SRC, NOS3, MAPK14, ATM, ATR, PGR, and CDK1. Collection of the SARS-CoV-2–related genes contained AR, CDK2, EGFR, ERBB2, GSK3B, HSP90AA1, JAK1, MET, MAPK1, and ROCK2. These included receptors, kinases, apoptosis, and cell cycle–related genes. They are all tumor-related in the conventional sense, but as we detailed earlier, SchB’s hepatoprotective mechanism involved protein kinase activation, apoptosis, and heat shock proteins. In addition, numerous genes have been linked to viral infections. HSP90AA1 has been reported to participate in influenza A virus–induced autophagy (Wang et al., 2020). The transcriptional repression of the androgen receptor enhanceosome inhibited SARS-CoV-2 infection *in vitro* (Wang et al., 2021). CDK2 reportedly plays a role in phosphorylating HBV capsids to trigger nucleocapsid disassembly during viral infection (Liu et al., 2021). Therefore, hepatitis- or virus-related route can explain the great majority of these genes that are enriched in the cancer pathway. In the investigation that followed, core targets were HSP90AA and CDK2, which were proven to be linked to viral infection. Among these core targets, MAPK1 had docking results (binding energy at −5.55 kcal/mol), but no hydrogen bonding, and thus, it could not be visualized by PyMOL mapping. Although slightly different in the details of GO analysis, such as the cell cycle pathway, adenovirus_hepatitis was enriched primarily in spindle binding, whereas SARS-CoV-2_hepatitis was reflected in chromatin binding in addition to spindle binding. These two collections, however, were both associated with kinase activation or inhibition, cell membrane binding, cell cycle, and oxidative stress energy metabolic functions.

## Conclusion

We developed comprehensive network pharmacology to identify targets of SchB on adenovirus_hepatitis and SARS-CoV-2_hepatitis. They shared a high degree of commonality in terms of core targets and KEGG and GO enrichment analyses. SchB has long been known to have liver-protective properties and recently was discovered to have anti-COVID-19 properties. The novelty of this study is that we proposed for the first time that SchB also may protect against hepatitis of unknown origin during the COVID-19 pandemic, and we verified its probable mechanism. This study provides a basis for further research determining the molecular targets of SchB on acute hepatitis in children as well as other types of COVID-19-related hepatitis.

## Data Availability

The original contributions presented in the study are included in the article/Supplementary Material; further inquiries can be directed to the corresponding authors.
